# 

**Published:** 1873-03

**Authors:** 


### Books and Pamphlets Received.

Medical and Surgical History of the War of the Rebellion. Prepared in accordance with Acts of Congress, under the direction of Surgeon General Joseph K. Barnes, U. S. A. Part first, two volumes. Vol. I, Medical, Vol. II, Surgical. Washington: Government Printing Office.

The Science and Art of Surgery. Being a treatise on Surgical Injuries, Diseases and Operations. By John Eric Erichsen, Senior Surgeon to University College Hospital, and Holme, Professor of Clinical Surgery in University College, London. New edition, enlarged and carefully revised by the author. Philadelphia: Henry C. Lea, 1873. Buffalo: T Butler & Son.

The Pharmacopœia of the United States of America. Fifth Decennial Revision. By authority of the Medical Convention for Revising the Pharmacopœia, held at Washington, A. D., 1870. Philadelphia: J. B. Lippincott & Co., 1873. Buffalo: H. H. Otis.

Principes De Biologie Appliqués a la Médicine par le Doctuer Ch. Girard. Paris: J. B. Bailliere et Fils, 1872. Received through Wm. Wesley, Agent, Smithsonian Institution, London.

THE BUFFALO

### Medical and Surgical Journal

PUBLISHED MONTHLY,

Containing Original Articles, Reports of Medical Societies and Hospitals, Editorial, Reviews, Correspondence, Army News, etc., etc.; including the usual variety of Medical Periodical Publications. Specimen copies sent on application.

Address Buffalo Medical & Surgical Journal, Buffalo, N. Y.

TERMS OF SUBSCRIPTION, - - $3.00 PER YEAR, IN ADVANCE.

